# Automatic measurement of fetal anterior neck lower jaw angle in nuchal translucency scans

**DOI:** 10.1038/s41598-024-55974-x

**Published:** 2024-03-04

**Authors:** Yulin Peng, Yingchun Luo, Junyi Yan, Wenjuan Li, Yimin Liao, Lingyu Yan, Hefei Ling, Can Long

**Affiliations:** 1 Department of Ultrasonography, Hunan Provincial Maternal and Child Health Care Hospital, No. 53 Xiangchun Road, Changsha, 410008 Hunan China; 2 NHC Key Laboratory of Birth Defect for Research and Prevention, Hunan Provincial Maternal and Child Health Care Hospital, Changsha, 410133 Hunan China; 3 Department of Ultrasonography, Second Xiangya Hospital of Central South University, No. 139 Renmin Middle Road, Changsha, 410028 Hunan China; 4 Clinical Laboratory, Hunan Provincial Maternal and Child Health Care Hospital, No. 53 Xiangchun Road, Changsha, 410008 Hunan China; 5 School of Computer Science, Hubei University of Technology, No. 28 Nanli Road, Wuhan, 430068 Hubei China; 6 School of Computer Science and Technology, Huazhong University of Science and Technology, No. 1037 Luoyu Road, Wuhan, 430074 China

**Keywords:** Ultrasonography, Computer science, Information technology, Medical imaging, Biomarkers

## Abstract

This study aims at suggesting an end-to-end algorithm based on a U-net-optimized generative adversarial network to predict anterior neck lower jaw angles (ANLJA), which are employed to define fetal head posture (FHP) during nuchal translucency (NT) measurement. We prospectively collected 720 FHP images (half hyperextension and half normal posture) and regarded manual measurement as the gold standard. Seventy percent of the FHP images (half hyperextension and half normal posture) were used to fit models, and the rest to evaluate them in the hyperextension group, normal posture group (NPG), and total group. The root mean square error, explained variation, and mean absolute percentage error (MAPE) were utilized for the validity assessment; the two-sample *t* test, Mann–Whitney *U* test, Wilcoxon signed-rank test, Bland–Altman plot, and intraclass correlation coefficient (ICC) for the reliability evaluation. Our suggested algorithm outperformed all the competitors in all groups and indices regarding validity, except for the MAPE, where the Inception-v3 surpassed ours in the NPG. The two-sample *t* test and Mann–Whitney *U* test indicated no significant difference between the suggested method and the gold standard in group-level comparison. The Wilcoxon signed-rank test revealed significant differences between our new approach and the gold standard in personal-level comparison. All points in Bland–Altman plots fell between the upper and lower limits of agreement. The inter-ICCs of ultrasonographers, our proposed algorithm, and its opponents were graded good reliability, good or moderate reliability, and moderate or poor reliability, respectively. Our proposed approach surpasses the competition and is as reliable as manual measurement.

## Introduction

Nuchal translucency (NT), the most frequently utilized ultrasonographic soft mark, refers to the anechoic patches at fetal napes between the hyperechoic skin and hyperechoic subcutaneous soft tissue at the gestation age of 11^1/7^ to 13^7/7^ weeks^1,2^. Floods of studies have proved the clinical value of NT, which makes NT scan a standard component of early prenatal screening^3,4^.

Much work has been done on NT-related artificial intelligence (AI) studies. Most of the studies focused on NT region location or NT measuring process^5–16^, and a few on midsagittal plane discrimination^17–19^. Hardly any have involved the other NT measurement criteria^20,21^. However, no criterion can be disregarded as they form the foundation of NT measurement accuracy^2,22,23^. We take for example fetal head posture (FHP), which must maintain appropriate posture over NT measurement^24,25^. Otherwise, hypertension can cause an NT overestimation of up to 0.62 mm and hyperflexion an NT underestimation of at most 0.5 mm^2,22^.

U-net networks, which look like U’s, are full convolutional networks optimized by fully convolutional networks^26^. The U-type networks need a smaller dataset size and boast higher segmentation accuracy than other convolutional neural networks^27^. The vanilla U-net network compromises a down-sampling path (encoder) in the left of the network and an up-sampling path (decoder) in the right. The former is designed to collect the context information, and the latter to pinpoint the location. The former consists of several 3 × 3 convolutional networks and 2 × 2 max pooling layers. The active function, $f\left( x \right)$, utilizes ReLU^28^ and is defined with $\tau > 0$ as

1 $$f\left( x \right) = \left\{ {\begin{array}{*{20}l} {x,} \hfill & {\quad x > 0} \hfill \\ {\tau \left( {e^{x} - 1} \right),} \hfill & {\quad x \le 0} \hfill \\ \end{array} } \right..$$

The deconvolution halves the number of channels, and its result is then spliced with the corresponding feature map. The spliced feature map is next convolved with a 3 × 3 kernel. The last layer applies a 1 × 1 convolution to map the 2-bit feature vectors onto the output layer. Nonetheless, the adaptation of vanilla U-net to new scenarios consists of numerous degrees of freedom involving the exact architecture, preprocessing, training, and inference, whose choices depend on each other and considerably influence the overall performance^29^.

Generative adversarial networks (GANs), first proposed in 2014^30^, are an emerging technique for learning deep features without demanding large amounts of annotated training data. The GANs are typically split into two parts: the generator and the discriminator. Conditional GANs^31^, a sort of classical GAN model, are predecessors of many GANs, say adversarially learned inference (ALI)^32^ or bidirectional GANs (BiGANs)^33^. The ALI and BiGANs supply simple but effective extensions that introduce an inference network where the discriminators inspect joint (data, latent) pairs. The structures of these two kinds of networks are illustrated in Supplementary Fig. S1.

**Figure 1:**
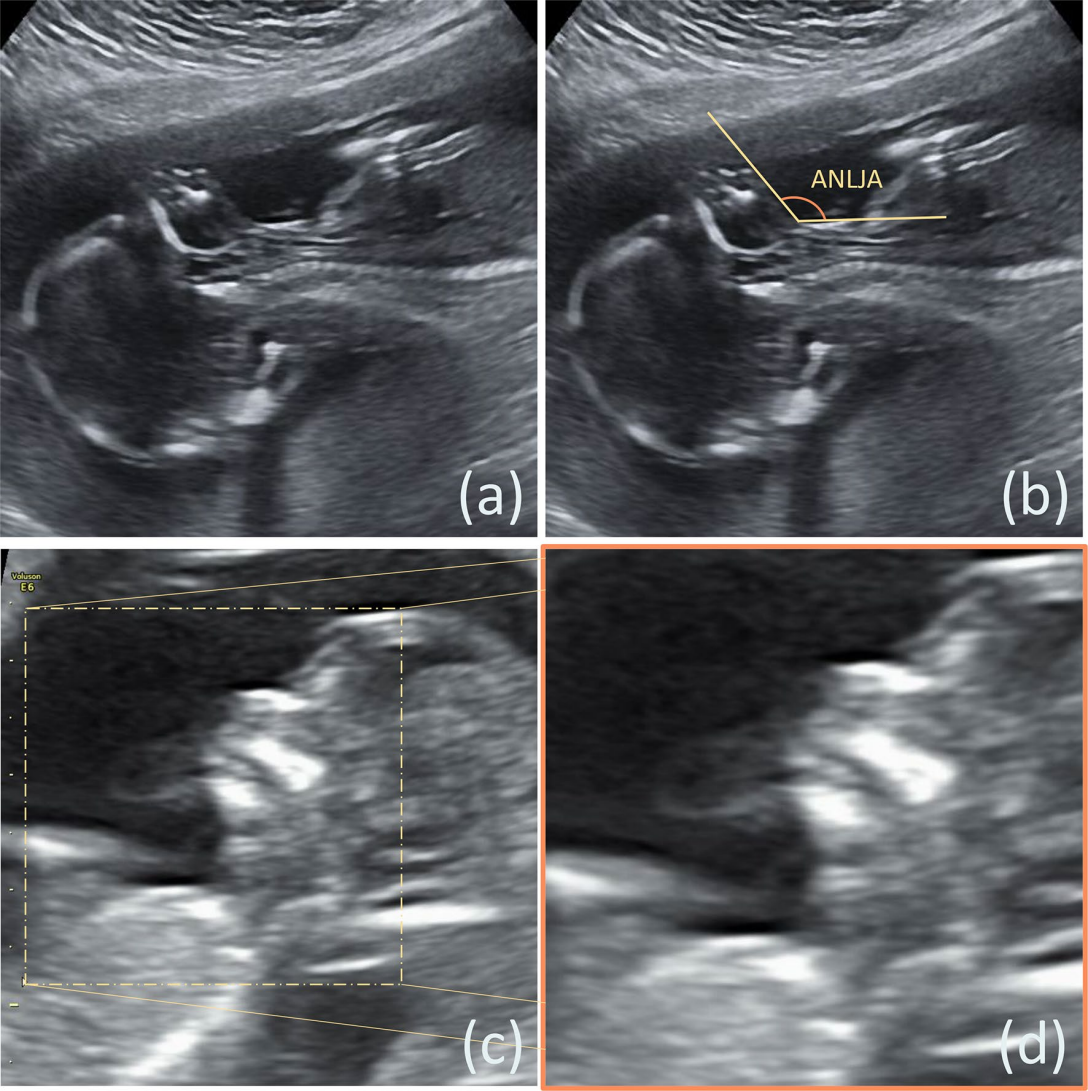
Data collection and preprocessing. (**a**) Original image of anterior neck lower jaw angle (ANLJA); (**b**) illustration of ANLJA measurement; (**c**) raw ANLJA image from an ultrasonoscope; (**d**) 400 × 400-pixel screenshot of ANLJA captured manually.

In this study, we provided an end-to-end prediction model based on a U-net-optimized GAN to forecast anterior neck lower jaw angle (ANLJA) (Fig. 1a), which was proposed to define FHP by Nuchal Translucency Quality Review (NTQR) Program. We then comprehensively evaluated all involved models on both validity and reliability.

## Results

### General information of study object

We collected an FHP image for each 720 singleton gravidas we recruited. Medians and interquartile ranges (IQRs) were adopted to describe objects’ general information because most variables conformed to non-normal distributions. The study objects’ general information is presented in Supplementary Table S1.

### Validity evaluation

**Figure 2:**
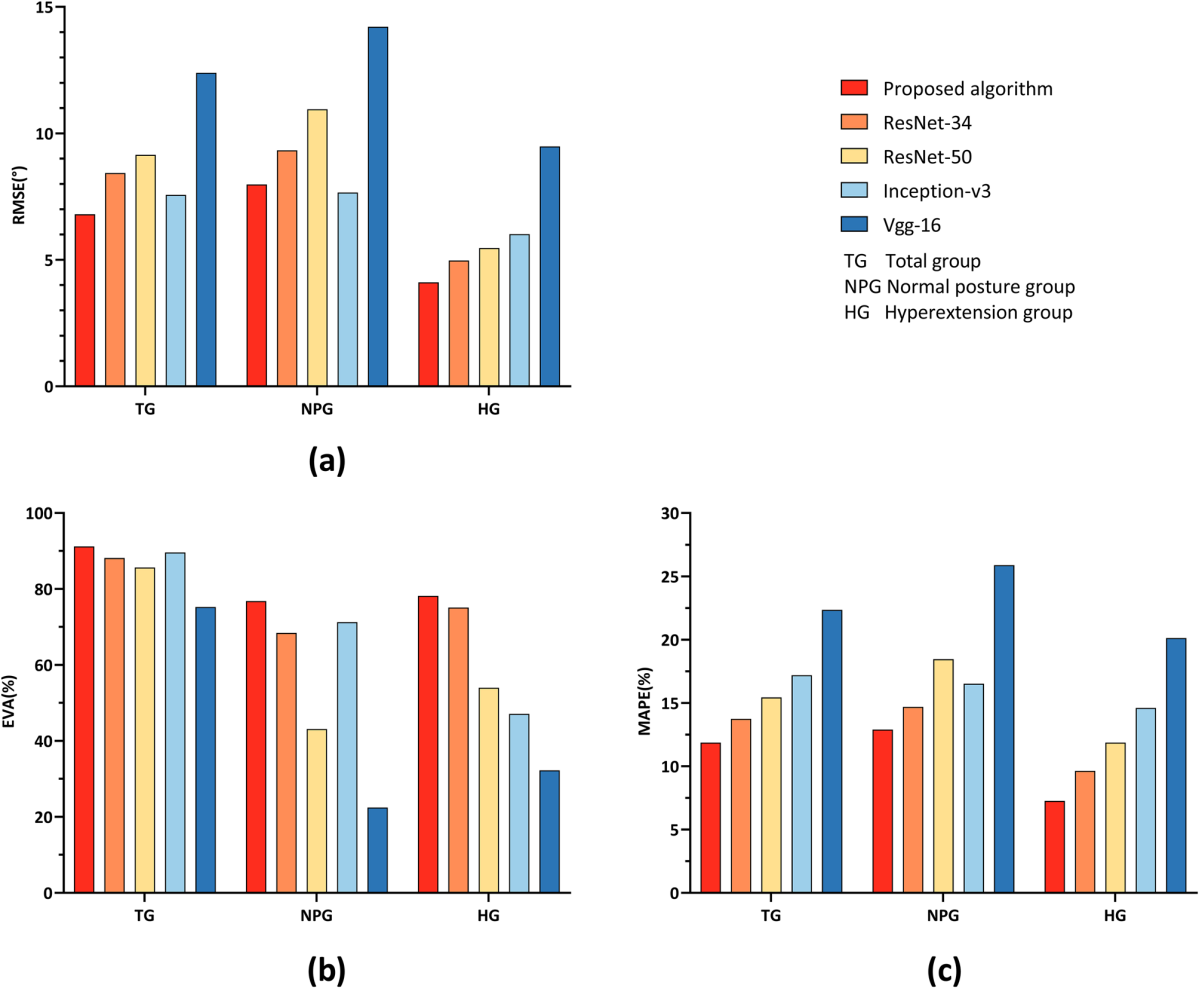
Comparison of different ANLJA prediction algorithms in validity assessment. (**a**), (**b**), and (**c**) are the bar charts of root mean square error (RMSE), explained variation (EVA), and mean absolute percentage error (MAPE), respectively. The smaller the RMSEs and MAPEs, the higher the performance; the bigger the EVA, the better the models operate. TG, NPG, and HG denote the Total group, Normal posture group, and Hyperextension group, respectively.

As shown in Fig. 2 and Table 1, the suggested algorithm surpassed all its competitors in all groups and indices, apart from the mean absolute percentage error (MAPE), where the Inception-v3 outperformed ours in the normal posture group (NPG).

**Table 1:** Comparison of ANLJA prediction models concerning validity.

Models	Total group			Normal posture group			Hyperextension group		
	RMSE (°)	EVA (%)	MAPE (%)	RMSE (°)	EVA (%)	MAPE (%)	RMSE (°)	EVA (%)	MAPE (%)
Proposed algorithm	5.21	93.43	10.85	6.88	78.37	13.89	4.50	79.62	7.89
ResNet-34	7.92	86.57	14.14	8.62	65.19	16.53	5.11	74.31	10.54
ResNet-50	8.68	83.63	16.88	9.77	48.55	19.29	6.89	59.14	12.48
Inception-v3	7.27	88.91	18.36	7.79	71.67	17.74	7.23	48.75	15.40
Vgg-16	11.54	75.72	23.73	13.21	26.41	26.17	10.42	42.61	20.51

*ANLJA* anterior neck lower jaw angle, *RMSE* root mean square error, *EVA* explained variation, *MAPE* mean absolute percentage error.

### Reliability evaluation

**Figure 3:**
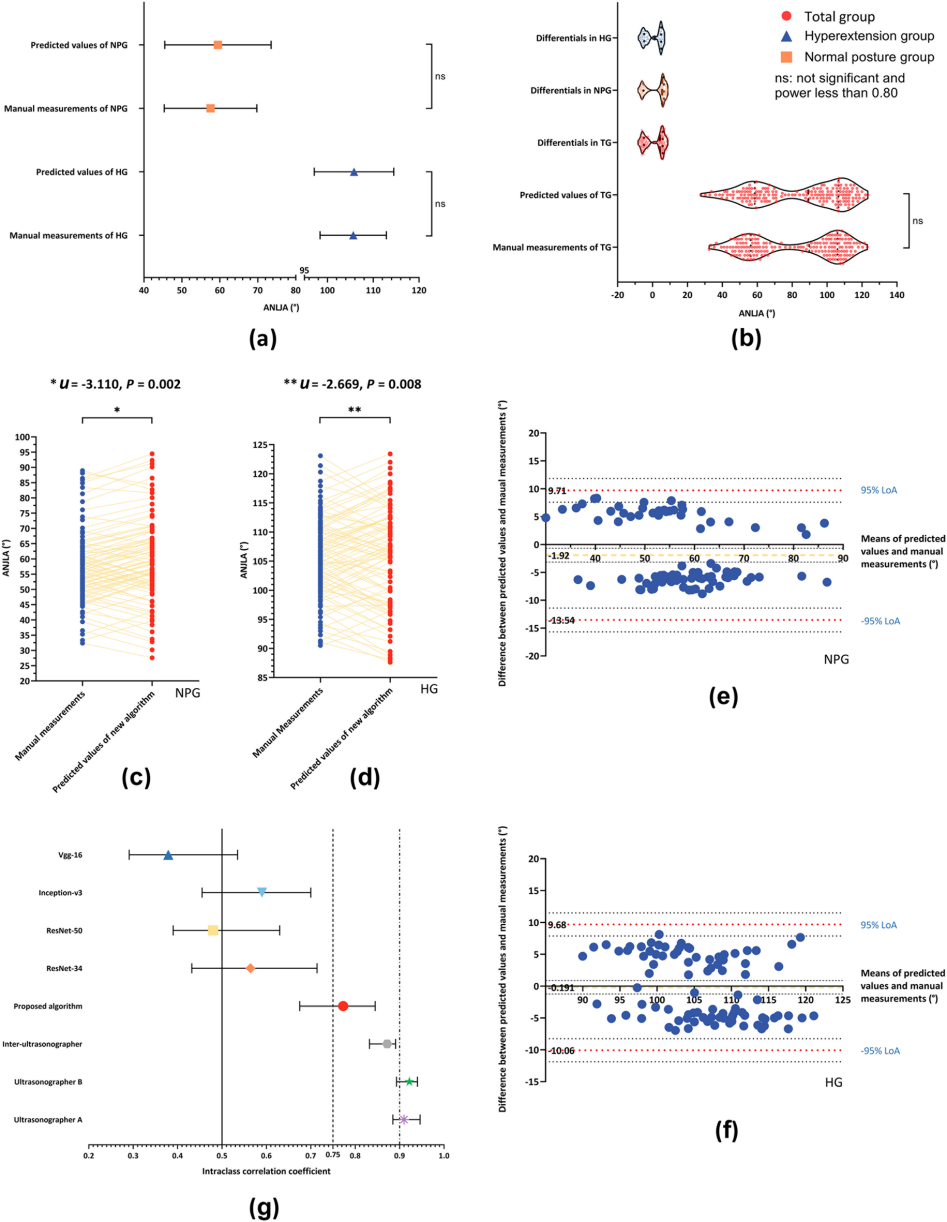
Comparison of algorithms and ultrasonographers in reliability assessment. (**a**) Group-level comparison of mean values of suggested method and manual measurement with two-sample* t* test (standard deviation bars). (**b**) Group-level comparison of medians of suggested method and manual measurement with Mann–Whitney* U* test and illustration of distributions of differentials between suggested method and manual measurement; (**c**) and (**d**) Personal-level comparison of suggested method to manual measurement with Wilcoxon signed-rank test. (**e**) and (**f**) Bland–Altman plots for reliability assessment between proposed algorithm and manual measurement. (**g**) Reliability evaluation for algorithms and ultrasonographers by intraclass correlation coefficient (ICC) with 95% confidence intervals. Poor reliability: ICCs below 0.50; moderate reliability: ICCs from 0.50 to 0.75; good reliability: ICCs between 0.75 and 0.90; excellent reliability: ICCs above 0.90. Two ultrasonographers’ ICCs are intra-ICCs, and the rest ICCs are inter-ICCs. NPG, Normal posture group; HG, Hyperextension group; TG, Total group; LoA, limit of agreement.

As indicated in Fig. 3a,b, and Table 2, we could not provisionally confirm any significant difference between the means of the predicted values of our new approach and the ground truth in the NPG and hyperextension group (HG). Neither could we temporarily when comparing the medians of the predicted values of our new approach to their ground truth in the total group (TG).

**Table 2:** Group-level comparison of proposed algorithm and manual measurement (gold standard) [Median (IQR), °].

Group	Manual measurements	Predicted values	Difference^a^	Statistics (*P* value)^b^
Normal posture group^*^	55.51 (49.39, 61.45)	58.39 (51.22, 65.70)	5.50 (− 5.23, 6.35)	− 1.080 (0.283)
Hyperextension group^$^	106.04 (101.44, 110.60)	106.57 (99.73, 112.33)	1.77 (− 4.97, 4.94)	− 0.172 (0.864)
Total group^#^	89.32 (55.48, 106.12)	89.16 (58.37, 106.93)	4.13 (− 5.00, 5.83)	15,573.50 (0.523)

^a^Differentials between predicted values and paired manual measurements; ^b^Power of test below 0.80.

^*^Values of the normal posture group follow a normal distribution; the mean and standard deviation of manual measurements are 56.18° and 10.87°, respectively; those of predicted values are 58.10° and 12.91°; the two-sample *t* test was used to determine the difference between groups.

^$^Values of the hyperextension group follow a normal distribution; the mean and standard deviation of manual measurements are 105.96° and 6.66°, respectively; those of predicted values are 106.15° and 8.22°; the two-sample *t* test was used to determine the difference between groups.

^#^Values of the total group follow a non-normal distribution; the Mann–Whitney *U* test was utilized to determine the difference between groups.

As seen in Fig. 3c and d, there is a significant difference between predicted values and their own ground truth in both NPG and HG.

The mean errors between the predicted values of our new method and the ground truth, as well as the limit of agreement (LoA), were illustrated in Fig. 3e and f. The Bland–Altman plots indicated that all points remained within the domain of [− 95% LoA, + 95% LoA]. It means no significant difference between the reliability of our novel method and the gold standard (manual measurement).

As presented in Fig. 3g, the two ultrasonographers’ intra-intraclass correlation coefficients (intra-ICCs) were graded good or excellent reliability, and their inter-intraclass correlation coefficients (inter-ICCs) good reliability. The inter-ICC of our suggested algorithm was ranked as good or moderate reliability, and the other algorithms as moderate or poor reliability.

### Ablation analysis

As described in Table 3, our proposed method (U-net + Wasserstein generative adversarial network with a gradient penalty (WGAN-GP) + multi-level multi-scale receptive field residual modular block (MMRFRMB)) is the most effective one with a root mean square error (RMSE) of 5.21°, an explained variation (EVA) of 93.43%, and a MAPE of 10.85%. The combination of U-net, BiGAN, and MMRFRMB is the second best, and that of U-net and WGAN-GP the third best. The model containing only U-net performed worst in the ablation test.

**Table 3:** Ablation analysis of the proposed method by testing data set of the total group.

Method				RMSE (°)	EVA (%)	MAPE (%)
U-net	WGAN-GP	BiGAN	MMRFRMB			
√	√	×	√	**5.21**	**93.43**	**10.85**
√	×	√	√	8.13	84.2	15.97
√	√	×	×	9.09	85.26	17.39
√	×	×	×	10.77	80.01	21.62

*WGAN-GP* Wasserstein generative adversarial network with a gradient penalty; multi-level multi-scale receptive field residual modular block, *RMSE* root mean square error, *EVA* explained variation, *MAPE* mean absolute percentage error.

The best results are shown in bold.

## Discussion

Most NT-related AI studies deal with narrow NT location or measurement problems, and only a few handle the issues of NT measurement criteria. Nevertheless, it is vital to ascertain whether an NT image follows all criteria and which criterion the NT image violates. Firstly, the violation causes measurement inaccuracies in all probability^2,22,23^. Secondly, it is necessary to identify which criterion or criteria have been broken in real-time NT measurement if we wish ultrasonoscope robots to obtain standard NT images and determine precise NTs automatically. That is because different situations require different solutions: we should shift non-midsagittal planes to midsagittal ones but wait for hyperextension or hyperflexion fetuses to revert to normal FHP.

We concentrated on the FHP scenario owing to the scarcity of FHP studies pertinent to NT-related AI research^17–19,34^. NTQR’s quantified definitions were adopted to prevent ambiguity because theirs are more unambiguous and easier to accept by novices than other ambiguous editions^24^. After careful consideration, we volunteered first to handle the ANLJA prediction problem to lay the groundwork for automatic FHP identification.

Our new approach boasts the following advantages contrasted with traditional deep learning-based models:

First, an end-to-end prediction algorithm for ANLJA was designed under an optimized U-net structure, which could precisely identify the two sides of ANLJAs and cope with relatively small datasets in medical image segmentation tasks.

Second, the receptive field blocks with small corns (RFBs) could not only efficiently diminish the parameter number and time complexity but also extract more details with their small cores. These enable the RFBs to abstract multi-scale features with minimized time complexity.

Third, each level of a receptive field dense block (RFDB) could receive features from the last RFDB, which forwarded the extractive capability of the local residual learning (LRL). The RAM was a hybrid attention mechanism composing channel and spatial attention. The former was used to learn attention to retrieve high-frequency information, and the latter was utilized to distinguish between channels to concentrate on low-frequency information.

Fourth, the MMRFRMB and receptive field residual modular blocks (RFRMB) deployed a long skip connection (LSC) and short skip connection (SSC), respectively. These made it easier for our model to bypass low-frequency information, further the LRL, and focus on data with channel attention.

Last but not least, we applied the discriminator network derived from the WGAN-GP and then executed the regularization to the Lipschitz-1 by making the constraint conditions the penalty terms of object functions. This discriminator network could remarkably boost the training effect and stability of GAN and resolve the slow convergence problem in the original Wasserstein generative adversarial network.

We compared our novel approach to four traditional deep learning models in the validity assessment by means of the RMSE, MAPE, and EVA.

The suggested method boasted the lowest RMSE in the TG and HG and the second lowest RMSE in the NPG. Meanwhile, the proposed approach fared best in all groups regarding the EVA and MAPE (Fig. 2 and Table 1).

Although the *P* values exceeded the significant level in all groups in the group-level comparison, all power of test were below 80% (Fig. 3a,b, and Table 2). We can only temporarily confirm that there is no significant difference between the two methods at group levels unless the sample size is expanded to such a point where the power of test reaches 80%^35^. Nonetheless, we need not do that despite the uncertainty because practitioners are more concerned with how closely different approaches correspond to one another at personal levels. Therefore, using the paired *t* test or Wilcoxon signed-rank test is more reasonable in the Reliability evaluation^36^.

We conducted the personal-level comparison utilizing the Wilcoxon signed-rank test since the difference between the suggested approach and manual measurement showed non-normal distributions in all groups (Fig. 3b). As illustrated in Fig. 3c and d, there are significant differences between the two methods in both groups.

We visualized the reliability assessment of the proposed algorithm and the manual measurement through Bland–Altman plots. All points fell between the upper and lower LoAs, which indicates no difference between the two methods in terms of reliability (Fig. 3e,f).

We also exploited the intra-ICCs to evaluate the intra-rater reliability between the ultrasonographers and inter-ICCs to assess the inter-rater reliability between the ultrasonographers and algorithms. The ultrasonographers’ intra-ICCs and inter-ICCs were graded good reliability or higher, which is common in well-designed and well-executed research^37^. The suggested algorithm was graded moderate or good reliability, higher than the others. In other words, the proposed algorithm surpassed all its rivals in Reliability evaluation.

In addition, we executed ablation analyses of the proposed method by the testing data set of the TG. Our proposed method (U-net + WGAN-GP + MMRFRMB) surpassed all the other competitors, which means the combination of U-net, WGAN-GP, and MMRFRMB boasts high effectiveness in the prediction of ANLJA.

The major limitation of our research was the relatively small sample size and mono-center design. However, the impact should be small. First, the proposed algorithm performed better than traditional ones statistically, according to our results, despite the sample size limitation. Second, our hospital is a national regional medical center with two campuses in Changsha (provincial capital) and numerous cooperating agencies all over Hunan province^38^.

Our near-term expectation of this research was to present a model dealing with an NT measurement criterion—fetal head posture, which had been usually neglected. Our long-term expectation was to lay a solid foundation for NT image quality control and further fully automatic NT measurement.

In conclusion, the differentiation of NT criteria is crucial for NT image quality control and further fully automatic NT measurement. Following NTQR’s quantified definitions of FHP, we provide a U-net-optimized end-to-end GAN for ANLJA prediction, which lays the groundwork for automatic FHP discrimination. The generative network composes numerous innovative customized structures, such as the RFBses, RFDBs, RFRMs, RFRMBs, and MMRFRMBs; the discriminator network is derived from the WGAN-GP. A series of rigorous tests, including validity, reliability, and ablation assessment, demonstrate that our cutting-edge algorithm is superior to all its rivals in ANLJA forecast and as dependable as manual measurement.

## Materials and methods

The study was conducted in accordance with the Declaration of Helsinki, STARD^39^, GRRAS^40^, and Recommendations for Reporting Machine Learning Analyses in Clinical Research^41^. All experimental protocols of this cross-sectional study have been approved by the ethics committee of the Hunan Provincial Maternal and Child Health Care Hospital (HPMCHCH) of the University of South China. Written informed consent has been acquired from all enlisted gravidas in this study.

### Patients

We prospectively and randomly recruited in this research a total of 720 gravidas who received an NT scan at the HPMCHCH between October 2019 and December 2021.

**Figure 4:**
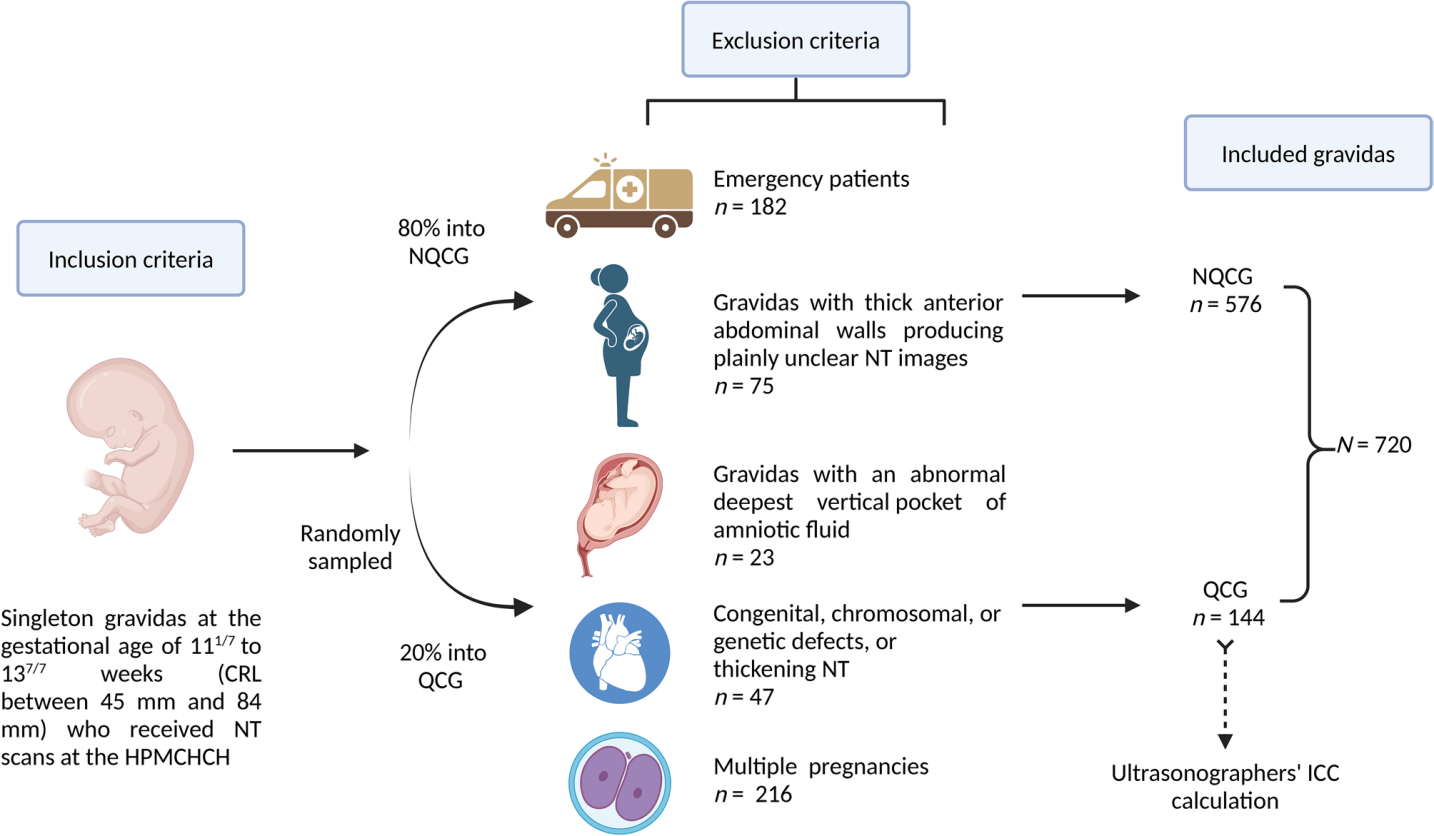
Flowchart of inclusion, exclusion, and quality control sampling. HPMCHCH, Hunan Provincial Maternal and Child Health Care Hospital; QCG, quality control group; NQCG, non-quality control group; NT, nuchal translucency; ICC, intraclass correlation coefficient.

The inclusion criteria for the candidates were singleton gravidas at the gestational age of 11^1/7^ to 13^7/7^ weeks (crown-lump length between 45 and 84 mm). The exclusion criteria for the candidates were emergency patients, multiple pregnancies, gravidas with an abnormal deepest vertical pocket of amniotic fluid or thick anterior abdominal walls producing plainly unclear NT images, or fetuses with chromosomal, genetic, or congenital defects or thickening NT (Fig. 4).

### Image collection

Pieces of Voluson E8 and E6 (General Electric) with RAB6-D probes (2.0–6.0 MHz) were utilized to collect FHP images after careful fine-tuning.

All FHP images were gathered when enlisted gravidas received their NT scans in the supine position by certified experienced sonographers. Appropriate FHP images were collected in brightness mode and stored in an ultrasound workstation as static images. Collected FHP images shall fulfill the following conditions: images should be clear; fetuses should occupy over half of the image area; fetal heads should stay in the midsagittal plane approximately^25^.

### Measurement and preprocessing

All ANLJAs were measured by two ultrasonographers using the caliper function of ultrasonoscopes (Fig. 1b). Their manual measurements served as the ground truth. Moreover, 20% of all images were randomly sampled for the quality control group (QCG) to assess the ultrasonographers’ inter-rater and intra-rater reliability, while the rest to the non-quality control group (NQCG). The images of QCG were evaluated twice by each of the aforementioned observers. Following NTQR’s definitions, a third party categorized images with the ground truth between 0° and 90° as normal posture, images with the ground truth above 90° as hyperextension, and images with the ground truth close to 0° as hyperflexion^25^. We only deployed hyperextension and normal posture images since the hyperflexion ones were unsuitable for ANLJA prediction.

We prospectively obtained a total of 720 clear FHP images, half of which were in normal posture and the other half in hyperextension. We manually screenshotted the regions of interest containing ANLJAs from raw ultrasound images and stored them as 400 × 400-pixel images using the ImageJ (Fig. 1c). These 400 × 400-pixel screenshots (Fig. 1d) and their corresponding ground truth comprised the entirety of our data set.

According to the ground truth of ANLJAs, we classified all images into the NPG and HG, each of which contained 360 images. The images from the aforesaid groups were then randomly divided into the training set and testing set individually at a ratio of 7:3 (Supplementary Table S2). The NPG and HG composed the TG.

### U-net-optimized Generative Adversarial Network

We demonstrated the overall structure of our end-to-end ANLJA prediction algorithm based on U-net-optimized GAN, where batch normalization was eliminated to increase training speed and decrease time complexity.

**Figure 5:**
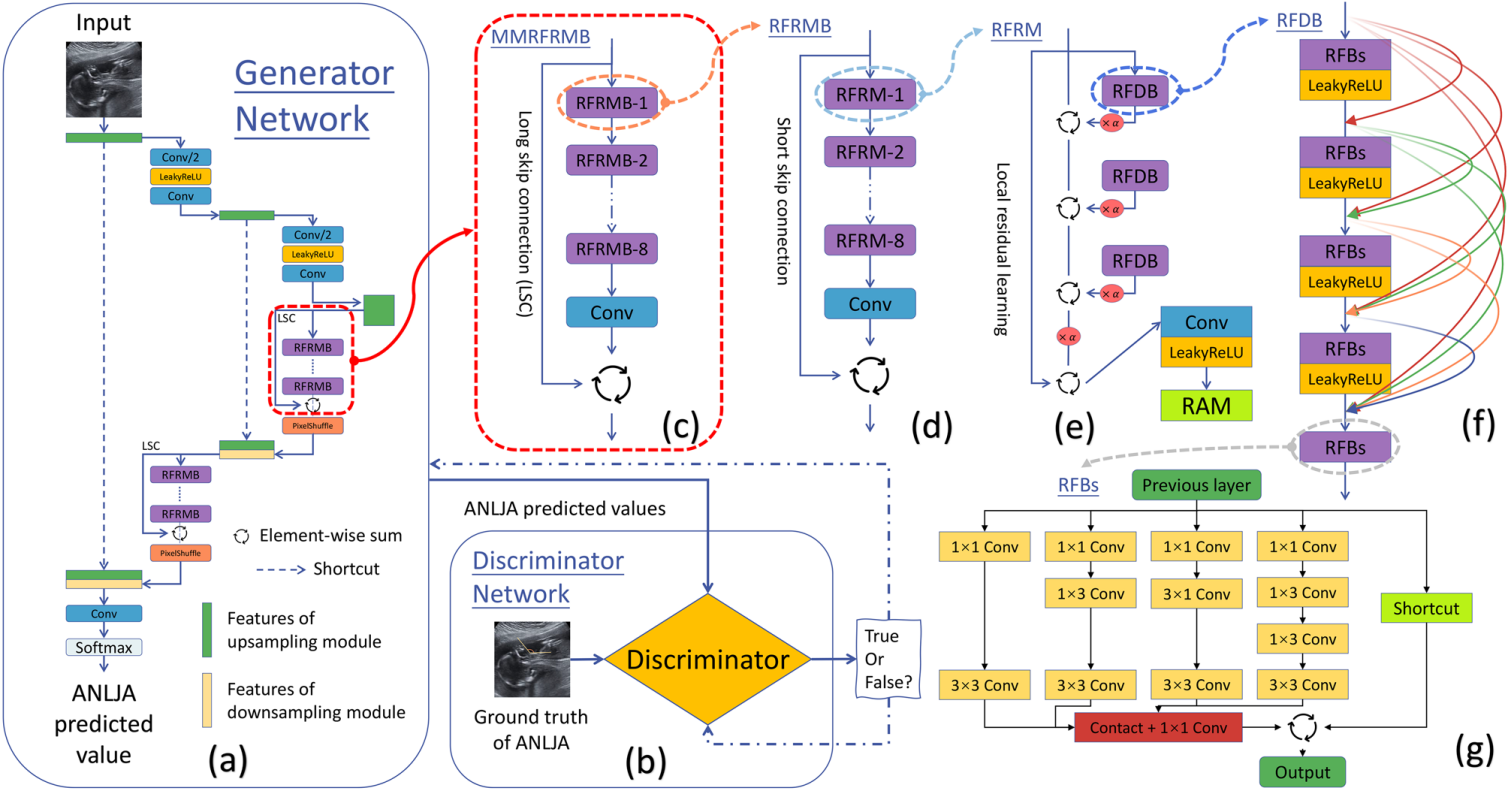
Structure of proposed ANLJA prediction algorithm. (**a**) Structure of U-net-based generator network; (**b**) Structure of discriminator network derived from Wasserstein generative adversarial network with a gradient penalty; (**c**) Structure of multi-level multi-scale receptive field residual modular block (MMRFRMB); (**d**) Structure of receptive field residual modular block (RFRMB); (**e**) Structure of receptive field residual module (RFRM); (**f**) Structure of receptive field dense block (RFDB); (**g**) Structure of receptive field block with small cores (RFBs). LSC, long skip connection; RAM denotes hybrid attention mechanism; α stands for feature weight.

The U-net-based generative network (Fig. 5a) featured two MMRFRMB (Fig. 5c) capable of extracting more texture features and advancing the model.

An MMRFRMB consisted of eight RFRMB, one convolutional layer, and one LSC. Input data through the LSC were integrated with the other processed by the eight RFRMBs and the subsequent convolutional layer. Next, the infused data underwent an element-wise sum operation.

The RFRMB (Fig. 5d) had a similar topology to the MMRFRMB, except that the LSC was replaced by an SSC. Noteworthily, batch normalization was removed from RFRMBs.

As depicted in Fig. 5e, input data were separated into three sections. The first portion was crunched by LRL directly, while the remainder were crunched numerous times by RFDB, feature weight α, and element-wise sum operation. All processed data fused together, underwent a convolutional layer and LeakyReLU function, and finally went through an attention mechanism RAM. The RAM was composed of both spatial and channel attention.

An RFDB (Fig. 5f) contained five RFBs and four LeakyReLU activation functions where the RFBses alternated with the LeakyReLU functions. An RFDB could be grouped into three sections according to the functions: contiguous memory, LRL, and local feature fusion (LFF). The contiguous memory sent the states of the last RFDB to every RFBs of the current RFDB, and the LFF merged the previous states and RFBses. The LRL was designed for the output of the last RFDB, and the LFF to promote information flow and network representation.

The RFBses (Fig. 5g) were modified from the original receptive field blocks by replacing big cores (e.g. 3*3 and 5*5) with small cores (e.g. 1*1, 1*3, and 3*1).

As to the discriminator network (Fig. 5b), we used the discriminator in the WGAN-GP^42^ with its batch normalization deleted (Supplementary Table S3).

### Model evaluation

Validity is the extent to which the predicted results closely match the actual ones^36^. We regarded the manual measurements as ground truth^43^. In addition, we employed the three most often utilized indices in angle prediction to evaluate the validity of our model and traditional AI algorithms. These indices were EVA, RMSE, and MAPE (Supplementary Equations S1–S5)^44^. Following their definitions, the smaller the RMSEs and MAPEs, the higher the performance; the bigger the EVA, the better the models operate.

Reliability is the consistency of obtaining identical results from identical objects under identical circumstances^36^. We considered the manual measurements as ground truth and utilized the two-sample *t* test, Mann–Whitney *U* test, Wilcoxon signed-rank test, Bland–Altman plots, and ICCs to evaluate the reliability.

The two-sample *t* test and Mann–Whitney *U* test are usually deployed to establish differences between algorithms at group levels. If both ground truth and predicted values of our new method followed normal distributions, we would compare them at group levels utilizing the two-sample *t* test. If conversely, we would use the Mann–Whitney *U* test to compare them. Moreover, if there was no significant difference between them, we would determine whether the power of test exceeded 80%^35^.

Practitioners care more about the difference between approaches to identical samples. Thus, applying the paired *t* test or Wilcoxon signed-rank test is more reasonable^35,36^. Both methods considered the difference between predicted values and their corresponding actual ones as a new variable. When the new variable shows a normal distribution, we should use the paired *t* test to compare zero and the mean of this new variable. Contrariwise, we should use the Wilcoxon signed-rank test to compare zero and the median of the new variable.

When assessing the reliability of two methods, scientists always use Bland–Altman plots to visualize the dissimilarity. If all points lie in the domain of − 95% LoA to + 95% LoA, it means there is no significant difference between these two methods in terms of reliability^45^. We assigned the differentials between ground truth and the predicted values of our new approach to ***y*** and their means to ***x*** to plot the points. We then marked the means of errors, LoAs, and their 95% confidential intervals with different types of lines.

The intra-ICC was used to quantify the two ultrasonographers’ intra-rater reliability with the QCG data; the inter-ICC to quantify their inter-rater reliability with their first QCG measurements. Similarly, the inter-ICC could be used to determine the reliability of algorithms (towards ground truth) since each algorithm could be considered an independent rater.

Additionally, we conducted ablation analyses with the testing set data of the TG to prove the effectiveness of our proposed approach for ANLJA prediction. The U-net served as the baseline. We later selected one of WGAN-GP/ BiGAN/ no GAN and one of MMRFRMB/ no MMRFRMB to form comparative items under the condition of the U-net.

### Statistical analyses

We deployed the RStudio 2023.03.2 + 454 (posit.co), PASS 2022 (NCSS Statistical Software), and Biorender.com for statistical analysis and visualization. We employed the Q–Q plot and Shapiro–Wilk test for the normality test^46^. Means and standard deviations were applied to describe normal distributional indices, whereas medians and IQRs were used to characterize non-normal distributional ones.

The two-sample *t* test is usually used for group-level comparisons under the assumption that both independent samples follow normal distributions. Otherwise, the Mann–Whitney *U* test should be utilized. The paired *t* test is typically employed to compare the predicted values and their matched ground truth if their difference shows normal distributions. If not, the Wilcoxon signed-rank test should be exploited. When the difference is not statistically significant, further action shall be conducted to verify whether or not the power of test exceeds 80%^35^.

The preconditions of the Bland-Atman plot, specifically the randomness, homoscedasticity, and normality of difference, were confirmed before we drew on it^47^.

The intra-ICCs were calculated with the absolute agreement, single measures, and two-way mixed effect model; similarly, the inter-ICCs were determined using the absolute agreement, single measures, and two-way random effect model. Finally, the point and interval estimations of ICCs were reported in accordance with the guidelines ^37,48,49^.

Additionally, all statistical analyses were conducted with the two-tailed test at a significant level of 0.05 without adjustment for multiple testing^37^.

### Ethical approval

Institutional Review Board approval was obtained.

### Consent to participate

Written informed consent was obtained from all subjects (patients) in this study.

### Supplementary Information

Supplementary Information 1.

Supplementary Information 2.

Supplementary Information 3.

Supplementary Information 4.

Supplementary Information 5.

## Data Availability

The data analyzed in this study will be available from the corresponding author on reasonable request after follow-up studies will have been conducted on these data.

## Abbreviations

AI: Artificial intelligence
ANLJA: Anterior neck lower jaw angle
BiGAN: Bidirectional generative adversarial network
EVA: Explained variation
FHP: Fetal head posture
GAN: Generative adversarial network
HG: Hyperextension group
HPMCHCH: Hunan Provincial Maternal and Child Health Care Hospital
ICC: Intraclass correlation coefficient
IQR: Interquartile range
LFF: Local feature fusion
LoA: Limit of agreement
LRL: Local residual learning
LSC: Long skip connection
MAPE: Mean absolute percentage error
MMRFRMB: Multi-level multi-scale receptive field residual modular block
NPG: Normal posture group
NQCG: Non-quality control group
NT: Nuchal translucency
NTQR: Nuchal Translucency Quality Review
QCG: Quality control group
RFDB: Receptive field dense block
RFRMB: Receptive field residual modular block
RFBs: Receptive field block with small corns
RMSE: Root mean square error
SSC: Short skip connection
TG: Total group
WGAN-GP: Wasserstein generative adversarial network with a gradient penalty
