# Supplementary material for: Automatic measurement of fetal anterior neck lower jaw angle in nuchal translucency scans
Source: Sci Rep. 2024 Mar 4;14:5351. doi: 10.1038/s41598-024-55974-x (PMC10912614; doi:10.1038/s41598-024-55974-x)
Supplement: Supplementary file 1 — Supplementary Information 1. [file 41598_2024_55974_MOESM1_ESM.pdf]

Supplementary Equations

The EVA is expressed by

$$EVA = 1 - \frac{Var(\hat{a}_i - a_i)}{Var(a)} \tag{1}$$

$$Var(\hat{a}_i - a_i) = \sum_{i=1}^{n} (\hat{a}_i - a_i)^2 \tag{2}$$

$$Var(a) = \sum_{i=1}^{n} (a_i - \bar{a})^2 \tag{3}$$

where Var, n, $\hat{a}_i$, a_i, and $\bar{a}$ stand for variance, the sample size, the predicted value of the ith sample, the actual value of the ith sample, and the mean of actual values, respectively.

The RMSE is described by

$$RMSE = \sqrt{\frac{1}{n} \sum_{i=1}^{n} (\hat{a}_i - a_i)^2} \tag{4}$$

in which n, $\hat{a}_i$, and a_i denote the sample size, the predicted value of the ith sample, and the actual value of the ith sample, respectively.

The MAPE is predicted by

$$MAPE = \frac{1}{n} \sum_{i=1}^{n} \left| \frac{\hat{a}_i - a_i}{a_i} \right| \tag{5}$$

where n, $\hat{a}_i$, and a_i represent the sample size, the predicted value of the ith sample, and the actual value of the ith sample, respectively.
