# Supplementary material for: Automatic measurement of fetal anterior neck lower jaw angle in nuchal translucency scans
Source: Sci Rep. 2024 Mar 4;14:5351. doi: 10.1038/s41598-024-55974-x (PMC10912614; doi:10.1038/s41598-024-55974-x)
Supplement: Supplementary file 2 — Supplementary Information 2. [file 41598_2024_55974_MOESM2_ESM.pdf]

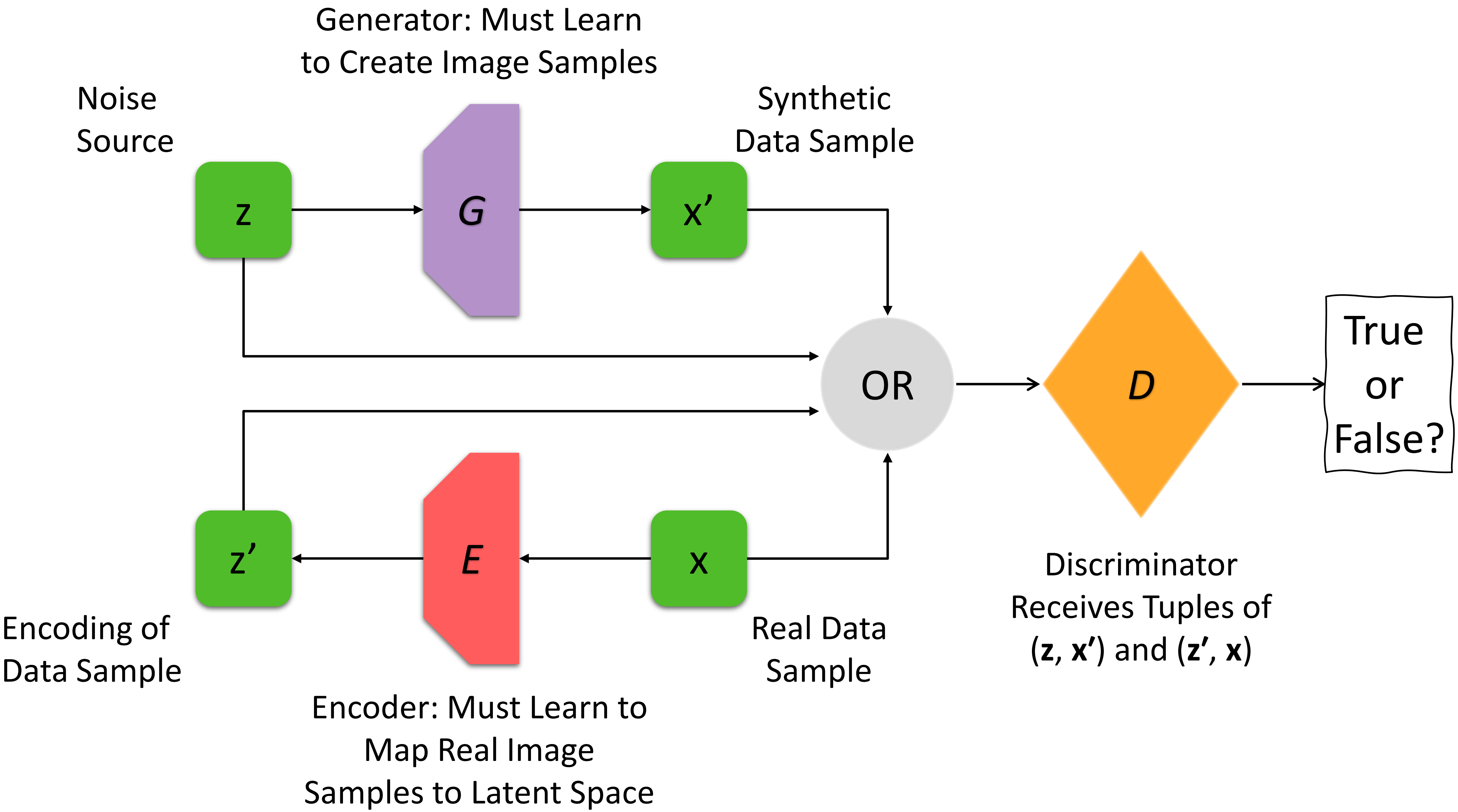

Supplementary Fig. S1 The ALI/BiGAN structure [33,34] compromises three networks. One serves as a discriminator, another maps the noise vectors from latent space to image space (decoder, described as a generator *G*), and the last (encoder, described as *E*) maps from image space to latent space.
