# Supplementary material for: Automatic measurement of fetal anterior neck lower jaw angle in nuchal translucency scans
Source: Sci Rep. 2024 Mar 4;14:5351. doi: 10.1038/s41598-024-55974-x (PMC10912614; doi:10.1038/s41598-024-55974-x)
Supplement: Supplementary file 3 — Supplementary Information 3. [file 41598_2024_55974_MOESM3_ESM.pdf]

Supplementary Table S1 General information of study objects

Parameters	Values
Gestational age, weeks, median (IQR)	13.3 (12.7-13.6)
Crown-lump length, mm, median (IQR)	64.9 (58.2-69.7)
Biparietal diameter, mm, median (IQR)	19.3 (17.1-22.8)
Nuchal translucency, mm, median (IQR)	0.13 (0.11-0.16)
Deepest vertical pocket, mm, median (IQR)	36.5 (31.7-43.3)
Age of gravidas, years, median (IQR)	28.3 (25.7-30.8)
BMI of gravidas, kg/m^2, median (IQR)	24.3 (21.7-27.6)
Thickness of anterior abdominal wall, mm, median (IQR) [a]	20.5 (16.9-23.8)
Assisted reproduction, n (%)	194 (26.9)
Plurigravida, n (%)	245 (34.0)
Cesarean delivery, n (%)	148 (20.6)

IQR, interquartile range; BMI, body mass index

[a] The thickness of anterior abdominal walls was measured at the point 1 cm on the left side to the navel
