# Supplementary material for: Automatic measurement of fetal anterior neck lower jaw angle in nuchal translucency scans
Source: Sci Rep. 2024 Mar 4;14:5351. doi: 10.1038/s41598-024-55974-x (PMC10912614; doi:10.1038/s41598-024-55974-x)
Supplement: Supplementary file 4 — Supplementary Information 4. [file 41598_2024_55974_MOESM4_ESM.pdf]

Supplementary Table S2 Arrangement of study objects

Sets	Normal posture group	Hyperextension group
Training set	270	270
Testing set	90	90
Total	360	360
