# Supplementary material for: Automatic measurement of fetal anterior neck lower jaw angle in nuchal translucency scans
Source: Sci Rep. 2024 Mar 4;14:5351. doi: 10.1038/s41598-024-55974-x (PMC10912614; doi:10.1038/s41598-024-55974-x)
Supplement: Supplementary file 5 — Supplementary Information 5. [file 41598_2024_55974_MOESM5_ESM.pdf]

Supplementary Table S3 Flowchart of Wasserstein generative adversarial network with a gradient penalty (WGAN-GP)

Default values of WGAN-GP: $\boldsymbol{\lambda}$, $\boldsymbol{\alpha}$, $\boldsymbol{n}_{\text{critic}}$, $\boldsymbol{\beta_1}$, and $\boldsymbol{\beta_2}$ are 10, 0.0001, 5, 0, 0.9, respectively.

$\boldsymbol{\lambda}$, $\boldsymbol{m}$, and $\boldsymbol{n}_{\text{critic}}$ are the gradient penalty factor, batch size, and iterations of evaluation indices generated by each generator, respectively. $\boldsymbol{\alpha}$, $\boldsymbol{\beta_1}$, and $\boldsymbol{\beta_2}$ are the hyper-parameters of adaptive moment estimation. $\boldsymbol{w_0}$ and $\boldsymbol{\theta_0}$ are the original evaluation parameter and the original parameter of the generator individually.

if $\boldsymbol{\theta}$ is inconvergent
to $\boldsymbol{t = 1, \dots, n}_{\text{cribic}}$
to $\boldsymbol{i = 1, \dots, m}$
real sample $\boldsymbol{x} \sim \mathbb{P}_{\boldsymbol{r}}$, potential variable $\boldsymbol{z} \sim \boldsymbol{p}(\boldsymbol{z})$, and random number $\boldsymbol{\epsilon} \sim \boldsymbol{U}[\mathbf{0}, \mathbf{1}]$
$\tilde{\boldsymbol{x}} \leftarrow \boldsymbol{G_\theta}(\boldsymbol{z})$
$\hat{\boldsymbol{x}} \leftarrow \boldsymbol{\epsilon x} + (\mathbf{1} - \boldsymbol{\epsilon})\tilde{\boldsymbol{x}}$
$\boldsymbol{L}^{(i)} \leftarrow \boldsymbol{D_w}(\tilde{\boldsymbol{x}}) - \boldsymbol{D_w}(\boldsymbol{x}) + \boldsymbol{\lambda}\left(\|\nabla_{\hat{x}}\boldsymbol{D_w}(\hat{\boldsymbol{x}})\|_2 - \mathbf{1}\right)^2$
end
$\boldsymbol{w} \leftarrow$ Adam $\left(\nabla_{\boldsymbol{w}} \frac{1}{m}\sum_{i=1}^{m} \boldsymbol{L}^{(i)}, \boldsymbol{w}, \boldsymbol{\alpha}, \boldsymbol{\beta_1}, \boldsymbol{\beta_2}\right)$
end
sample potential variables $\{\boldsymbol{z}^{(i)}\}_{i=1}^{m} \sim \boldsymbol{p}(\boldsymbol{z})$
$\boldsymbol{\theta} \leftarrow$ **Adam** $\left(\nabla_{\boldsymbol{\theta}} \frac{1}{m}\sum_{i=1}^{m} - \boldsymbol{D_w}(\boldsymbol{G_\theta}(\boldsymbol{z})), \boldsymbol{\theta}, \boldsymbol{\alpha}, \boldsymbol{\beta_1}, \boldsymbol{\beta_2}\right)$
end circulation
